# Evaluation of viral infection as an etiology of ME/CFS: a systematic review and meta-analysis

**DOI:** 10.1186/s12967-023-04635-0

**Published:** 2023-10-28

**Authors:** Jae-Hyun Hwang, Jin-Seok Lee, Hyeon-Muk Oh, Eun-Jung Lee, Eun-Jin Lim, Chang-Gue Son

**Affiliations:** 1 Korean Medical College of Daejeon University, 62, Daehak-Ro, Dong-Gu, Daejeon, Republic of Korea 34520; 2 Institute of Bioscience and Integrative Medicine, Daejeon University, 62 Daehak-Ro, Dong-Gu, Daejeon, Republic of Korea 34520; 3 Department of Korean Rehabilitation Medicine, College of Korean Medicine, Daejeon University, 176 Daedeok-Daero, Seo-Gu, Daejeon, Republic of Korea 35235; 4 Department of Integrative Medicine, Graduate School of Integrative Medicine, CHA University, 335 Pangyo-Ro, Bundang-Gu, Seongnam-city, 13488 Gyeonggi-Do Korea; 5 Research Center for CFS/ME, Daejeon Oriental Hospital of Daejeon University, 176 Daedeok-Daero, Seo-Gu, Daejeon, Republic of Korea 35235

**Keywords:** Myalgic encephalitis (ME), Chronic fatigue syndrome (CFS), Viral infection, Meta-analysis, Odds ratio, Etiopathogenesis, Disease causation

## Abstract

**Background:**

Myalgic encephalitis/chronic fatigue syndrome (ME/CFS) is a long-term disabling illness without a medically explained cause. Recently during COVID-19 pandemic, many studies have confirmed the symptoms similar to ME/CFS in the recovered individuals. To investigate the virus-related etiopathogenesis of ME/CFS, we conducted a systematic assessment of viral infection frequency in ME/CFS patients.

**Methods:**

We conducted a comprehensive search of PubMed and the Cochrane Library from their inception through December 31, 2022, using selection criteria of viral infection prevalence in ME/CFS patients and controls. Subsequently, we performed a meta-analysis to assess the extent of viral infections' contribution to ME/CFS by comparing the odds ratio between ME/CFS patients and controls (healthy and/or diseased).

**Results:**

Finally, 64 studies met our eligibility criteria regarding 18 species of viruses, including a total of 4971 ME/CFS patients and 9221 control subjects. The participants included healthy subjects and individuals with one of 10 diseases, such as multiple sclerosis or fibromyalgia. Two DNA viruses (human herpes virus (HHV)-7 and parvovirus B19, including their co-infection) and 3 RNA viruses (borna disease virus (BDV), enterovirus and coxsackie B virus) showed odds ratios greater than 2.0 compared with healthy and/or diseased subjects. Specifically, BDV exceeded the cutoff with an odds ratio of ≥ 3.47 (indicating a "moderate association" by Cohen’s d test) compared to both healthy and diseased controls.

**Conclusion:**

This study comprehensively evaluated the risk of viral infections associated with ME/CFS, and identified BDV. These results provide valuable reference data for future studies investigating the role of viruses in the causation of ME/CFS.

**Supplementary Information:**

The online version contains supplementary material available at 10.1186/s12967-023-04635-0.

## Introduction

Myalgic encephalomyelitis/chronic fatigue syndrome (ME/CFS) is a long-term disabling illness that is characterized by medically unexplained fatigue impairing daily life over 6 months [1]. According to the 2015 IOM, ME/CFS is characterized by complaints of several core symptoms, including post-exertional malaise (PEM), unrefreshing sleep, cognitive impairment and/or orthostatic intolerance [2]. A previous meta-analysis reported that the global prevalence of ME/CFS was approximately 0.89% according to the 1994 CDC criteria, which were the most frequently applied standards [3]. It has been estimated that there are 800,000 CFS patients in the USA [4].

One study found that 29% of ME/CFS patients were housebound, 27% were bedbound and 19% were unable to work at all [5]. Moreover, another study revealed that the most frequent cause of death in ME/CFS patients was suicide, corresponding to a 5–sevenfold higher rate among ME/CFS patients than among healthy subjects [6, 7]. Accordingly, there are urgent requirements for appropriate treatments, but no standard therapeutics have yet been approved [8]. Moreover, despite definite physical abnormalities in ME/CFS patients, no laboratory or objective diagnostic biomarkers have been established thus far [9]. These limitations are due to the undefined etiology and pathophysiology of ME/CFS [10]. Many proposed etiologies have been investigated, including viral infection, immune and/or neuroendocrine disturbance, decreased hypothalamic–pituitary–adrenal (HPA) axis activity, and abnormal cytokine secretion [11–13]. These hypotheses, however, failed to convincingly explain the etiology [14]. Additionally, studies of mitochondrial dysregulation, neuroinflammation, and abnormal upregulation of TGF-B have recently provided some insight into the pathophysiology of ME/CFS [11, 15].

On the other hand, the COVID-19 pandemic suggested a possible linkage between viral infection and ME/CFS because certain subjects with long COVID report symptoms similar to those of ME/CFS, including unrelieved fatigue, cognitive dysfunction and PEM [16]. One research group found that among 465 long-COVID patients, 58% met the 1994 CDC criteria for ME/CFS [17]. A large study comprising 3762 long-term CaOVID-19 patients across 56 countries showed a 56.8% prevalence of PEM, one of the key ME/CFS symptoms [18]. Another group reported that the clinical features of neuroinflammation overlapped between long COVID and ME/CFS, thus leading to cognitive dysfunction, unrefreshed sleep and fatigue [19]. These facts would indicate the opportunity to inspect the role of viral infection in the etiology or pathophysiology of ME/CFS. In fact, the name of ME (Myalgic Encephalomyelitis) derives from the sustained belief that central neural inflammation contributed to all viruses, since fatigue-dominant outbreaks, which have been proposed to be induced by viral infection, were initially named “benign encephalomyelitis” by JE Jelinek [20]. Another alternative term for ME/CFS—‘postviral fatigue syndrome’ (PVFS)—also suggests an association with viral infection [21]. In this background, understanding the role of viral infections in ME/CFS can imply the neuroinflammation views on ME/CFS, and possible following treatment.

Regarding the exploration of viral infection theory, we have carefully waited for long-term outcomes and consequences of COVID-19. Given that previous studies about linkage of viral infection and ME/CFS have conducted meta-analysis on only specific viral infection, we herein aim to comprehensively conducted meta-analysis on as many viruses as possible, and their extent to which viral infections contribute to ME/CFS by comparing ME/CFS patients with healthy and/or other diseased controls. This study is being conducted before we can obtain data from long-COVID subjects.

## Methods

### Data sources and keywords

The PubMed and Cochrane Library databases were searched from inception to December 31, 2022. The search keywords were ‘virus’, ‘Myalagic encephalitis’ and ‘Chronic fatigue syndrome’ [MeSH term]. We used the search terms “(virus) [All field] AND ((CFS) OR (Chronic fatigue syndrome) OR (ME) OR (Myalagic encephalitis)) [Title]. This systematic review was registered (PROSPERO registration number: CRD42021270498).

### Eligibility criteria

Articles were screened based on the following inclusion criteria: (1) clinical articles investigating viral infection from an etiological perspective, (2) studies involving both ME/CFS patients and control subjects, regardless of their health status, and (3) articles containing data on viral prevalence among ME/CFS patients and control groups. The exclusion criteria were as follows: (1) duplicate article, (2) article with main content unrelated to ME/CFS, (3) no full text exist or retracted article, (4) not clinical data (e.g., review), (5) no available viral infection prevalence data, (6) studies conducted within already viral-infected ME/CFS patients or controls, (7) studies without control group, and (8) not published in English.

### Review process and data extraction

The authors searched the databases for potentially eligible studies. The titles and abstracts of the retrieved studies were screened in accordance with the inclusion and exclusion criteria. Then, the full texts of potentially eligible studies were independently reviewed and cross-checked. We extracted the following data from each study: publication year, first author, country, study design, number of ME/CFS patients and control group, sex information (if possible), targeted virus, ME/CFS diagnostic case definition, viral detection method, and viral infection prevalence rate in the ME/CFS group and control groups.

### Assessment of study quality and heterogeneity

To assess the quality of each included study, we adapted the Newcastle Ottawa Scale (NOS) for nonrandomized studies [22]. Study quality was assessed by examining patient selection methods, comparability of groups, and assessment of outcome. The results are reported in Additional file 2: Table S1. Regarding assessment of the heterogeneity of studies, the ${I}^{2}$ statistic was used for healthy and diseased controls separately (Additional file 3: Table S2). The *I*^2^ value describes the probability of total variation across studies due to heterogeneity rather than chance or random error [23]. A *I*^2^ value of 50% reflects significant heterogeneity that is due to real differences in study populations, protocols, interventions, and outcomes.

### Meta-analysis for causality assessment of viral infection in ME/CFS

We divided data according to controls, healthy control group and diseased control group. Using Review Manager 5.3 software, meta-analyses were performed to assess the odds ratio of virus infection in ME/CFS patients by comparing them to both healthy control group and diseased control group, separately for each individual virus. And for the 3 different detection methods of viral infection—DNA/RNA viral genome using polymerase chain reaction (PCR) or reverse transcription (RT)-PCR, detection antibody titer (IgG or IgM positivity), and antigen detection—we separately calculated their odds ratios according to these detection methods using the Mantel–Haenszel method. These odds ratios were merged for each viral infection and used in meta-analyses. We employed a fixed-effect model for cases with heterogeneity less than 50% and a random-effects model for cases with 50% or greater heterogeneity. A p ≤ 0.05 indicated statistical significance. We also weighted studies based on sample size and the potential for publication and reporting bias was assessed using funnel plots and Egger’s test [24].

## Results

### Characteristics of the included studies

**Fig. 1:**
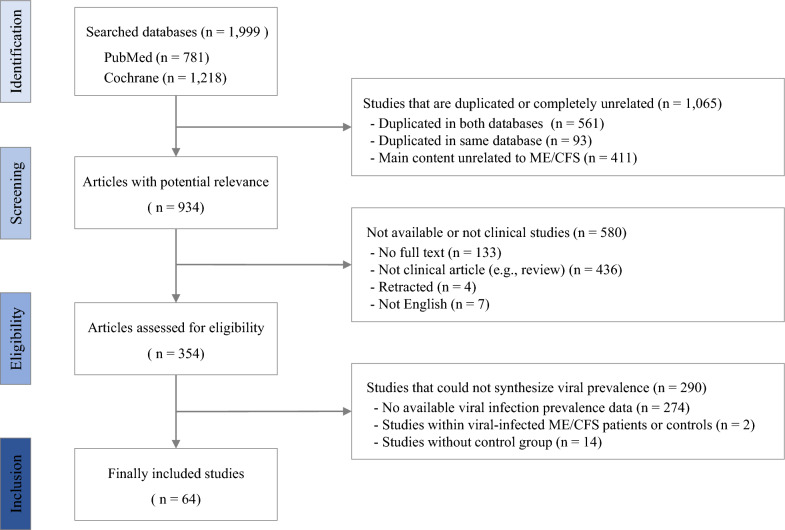
Flow chart of study selection. n: Number of study, ME/CFS: Myalgic encephalomyelitis/Chronic fatigue syndrome

Among 1999 articles initially identified from two databases, 64 studies met our study criteria (Fig. 1, Details in Additional file 2: Table S1). All the studies are case–control study. Regarding 18 species of the viruses (DNA virus 12, RNA virus 4, Retrovirus 2), a total of 4970 ME/CFS patients (male 807, female 1974 and unknown 2189) and 5584 control subjects (male 1165, female 1503 and unknown 2916) participated. The control groups included healthy subjects and individuals with one of 10 diseases, such as multiple sclerosis (7 studies) or fibromyalgia (5 studies) (Table 1). Seven studies (5 species of viruses) included ‘non-ME/CFS’ controls, but it is unclear whether these subjects were healthy or diseased.

**Table 1:** Summary of characteristics of included 64 studies

Items	N. of study	N. ME/CFS case	N. Control
			(Healthy or 10 Diseases)
Number of participants	64	4971 (77.6 ± 61.8)	5564 (87.2 ± 106.9)
Male/female^a^		807/1974	1165/1503
Enveloped virus			
*DNA virus*			
HHV family^b^	24	1694	1318 (5)
HHV-6	24	1694	1318 (5)
HSV-1	3	422	184 (2)
HSV-2	2	522	168 (2)
EBV	14	1301	1108 (5)
CMV	7	974	606 (4)
VZV	2	272	178 (2)
*RNA virus*			
BDV	3	321	338 (2)
Hepatitis C virus	1	36	14 (1)
*Retrovirus*			
XMRV	25	1579	2335 (9)
Retrovirus	8	240	363 (2)
Non-enveloped virus			
*DNA virus*			
Parvovirus B19	6	553	439 (1)
JC virus	1	22	22 (1)
BK virus	1	22	22 (1)
*RNA virus*			
Enterovirus	5	533	264 (3)
Coxsackie B virus	1	290	500 (1)
Detection method^c^			
DNA detection (PCR)	42	3035	4863
RNA detection (PCR)	10	800	913
Antibody detection	33	2755	5916
Antigen detection	5	414	281
CFS criteria^d^			
Fukuda	40	3559	–
Holmes	10	342	–
Canadian	8	745	–
Oxford	3	137	–
2015 IOM	1	166	–
Others	5	359	–
Unknown	3	362	–
Publication year			
1988–1999	23	1451	1668
2000–2009	11	636	477
2010–2022	30	2884	3440
Continent (N)			
Europe	28	2804	2802
North America	27	1688	1653
Asia	9	479	1109
Country (N)			
United States	25	1613	1588
United Kingdom	12	1654	1712
Japan	8	414	1014
Latvia	4	409	246
Sweden	3	312	317
Germany	3	114	199
The Netherland	2	108	119
Italy	2	101	124
Canada	2	75	65
China	1	65	85
Bulgaria	1	58	50
Belgium	1	48	35

^*^N Number, *HHV* Human herpes virus, *HSV* Human simplex virus, *EBV* Epstein barr virus, *CMV* Cytomegalovirus, *VZV* Varicella zoster virus, *BDV* Borna disease virus

^a^Only informed data are included

^b^HHV-1,2,3,5,6,7, HSV included except EBV(HHV-4) and CMV(HHV-5)

^c^Some articles used multiple methods

^d^Major context of CFS diagnostic criteria con be found in [53] (Lim et al.)

(RT)-PCR, measurement of antibody titer and/or virus antigen detection were used to detect viral infections for 10, 18, and 5 viruses, respectively (Tables 1, 2, 3). The results of the NOS showed that 55 studies (85.9%) scored 6 points or more, while the rest (9 studies) scored less than 6 points (Additional file 2: Table S1). From the assessment of heterogeneity, two data for Epstein-barr virus (EBV, healthy control) and enterovirus (diseased control) showed over 50% heterogeneity, while Egger test showed the probability of publication bias for data for 2 viruses (Additional file 1: Fig. S1, Additional file 3: Table S2).

**Table 2:** Odds ratios of DNA virus infection in ME/CFS patients

Infection	PCR		Antibody detection	
(Number. of study, PCR/Ab)	Number. of patients/controls	Odds ratio	Number. of patients/controls	Odds ratio
		[95% CI]		[95% CI]
Enveloped DNA virus				
HHV-1, 2, 3 (0/1)	None		Healthy (65/87)	0.93 [0.60, 1.45]
EBV (HHV-4) (6/8)	Healthy (374/256)	0.84 [0.54, 1.31]	Healthy (666/396)	1.26 [0.83, 1.91]
			MS (250/49)	1.81 [0.82, 3.99]
			FM (65/11)	1.20[0.13, 11.37]
			Non-ME/CFS (10/28)	Not estimable^*^
			CF(101/35)	1.06 [0.31, 3.56]
CMV (HHV-5) (2/5)	Healthy (80/72)	0.91 [0.40, 2.08]	Healthy (395/255)	0.70 [0.49, 1.01]
			MS (250/40)	0.52 [0.27, 1.03]
			FM (65/11)	0.29 [0.06, 1.47]
			Non-ME/CFS (165/34)	Not estimable
HHV-6 (13/11)	Healthy (768/510)	1.47 [1.10, 1.98]	Healthy (853/587)	1.42 [1.00, 2.01]
	CF (3/2)	3.00 [0.08, 115.34]	MS (250/40)	1.21 [0.39, 3.73]
	Non-ME/CFS (8/7)	0.83 [0.08, 8.24]	FM (65/11)	1.09 [0.21, 5.76]
HHV-7 (6/4)	Healthy (255/213)	1.67 [1.07, 2.60]	Healthy (228/217)	2.28 [1.39, 3.73]
			FM (65/11)	1.67 [0.40, 6.88]
HHV-8 (2/2)	Healthy (22/22)	Not estimable	Healthy (57/47)	0.58 [0.11, 3.14]
	Non-ME/CFS (8/7)	Not estimable		
HSV-1 (1/2)	Healthy (30/16)	1.07 [0.09, 12.81]	Healthy (272/128)	1.18 [0.77, 1.81]
			MS (250/40)	0.59 [0.30, 1.16]
HSV-2 (0/2)	None		Healthy (272/128)	1.27 [0.80, 2.00]
			MS (250/40)	0.97 [0.49, 1.91]
VZV (1/2)	Healthy (22/22)	Not estimable	Healthy (250/106)	0.58 [0.12, 2.79]
			MS (250/40)	0.78 [0.09, 6.37]
Non-enveloped DNA virus				
Parvovirus B19 (4/3)	Healthy (328/306)	5.55 [2.61, 11.79]	Healthy (366/309)	4.33 [1.22, 15.37]
JC virus (1/0)	Healthy (22/22)	Not estimable	None	
BK virus (1/0)	Healthy (22/22)	Not estimable	None	

*MS* multiple sclerosis (demyelinating disease in CNS), *FM* fibromyalgia (chronic widespread pain), *CF* chronic fatigue (over 6 months-fatigue)

Not estimable; Any infection was not detected either in ME/CFS group or control group, thus could not synthesize odds ratios

**Table 3:** Odds ratios of RNA virus, retro virus and co-infection in ME/CFS patients

Infection	PCR detection		Antibody detection	
(Number. of study, PCR/Ab)	Number. of patients/controls	Odds Ratio	Number. of patients/controls	Odds Ratio
		[95% CI]		[95% CI]
Enveloped RNA virus				
BDV (2/2)	Healthy (64/175)	3.82 [1.45, 10.03]	Healthy (176/33)	3.25 [0.83, 12.71]
			Non-ME/CFS (169/33)	12.93 [0.77, 217.38]
Hepatitis C virus (0/1)	None		Healthy (36/14)	1.23 [0.05, 31.87]
Non-enveloped RNA virus				
Enterovirus(4/2)	Healthy (76/76)	3.04 [0.12, 75.80]	Healthy (76/76)	0.93 [0.43, 2.00]
	ND (121/101)	1.46 [0.77, 2.75]	Non-ME/CFS (165/34)	17.36 [6.91, 43.58]
	Non-ME/CFS (54/31)	12.00 [1.37, 105.98]		
Coxsackie B virus (0/1)	None		Healthy (290/500)	6.15 [4.16, 9.09]
Enveloped retro virus				
XMRV (16/9)	Healthy (1232/1318)	1.79 [0.43, 7.52]	Healthy (734/1052)	1.05 [0.32, 3.44]
	MS (39/50)	Not estimable	Prostate cancer (100/67)	0.66 [0.09, 4.83]
	RA (97/122)	Not estimable	MS (36/112)	0.77 [0.08, 7.13]
	CF (61/6)	2.35 [0.12, 45.32]	Non-ME/CFS (170/395)	0.09 [0.01, 0.65]
	FM (65/55)	Not estimable		
	Transplants (32/26)	Not estimable		
	HIV (32/43)	Not estimable		
	Non-ME/CFS (276/84)	1.27 [0.06, 26.55]		
Other retrovirus (8/0)	Healthy (240/348)	2.14 [0.83, 5.48]	None	
	NMD (30/15)	Not estimable		
Co-infection				
HHV-6A, 6B (1/0)	Healthy (26/50)	5.94 [0.23, 151.07]	None	
HHV-6, 7 (2/1)	Healthy (83/74)	2.00 [0.95, 4.19]	Healthy (108/90)	19.29 [1.11, 334.01]
	CF (10/2)	3.46 [0.13, 90.68]		
HHV-7, Parvovirus B19 (0/1)	None		Healthy (108/90)	30.01 [1.77, 508.94]
HHV-6, 7, Parvovirus B19 (0/1)	None		Healthy (108/90)	7.79 [0.41, 146.74]

*ND* neurological disease (disorder of the nervous system), *MS* multiple sclerosis (demyelinating disease in CNS), *RA* rheumatoid arthritis (autoimmune disorder), *CF* chronic fatigue (over 6 months-fatigue), *FM* fibromyalgia (chronic widespread pain), *NMD* neurological/muscular disease

Not estimable; Any infection was not detected either in ME/CFS group or control group, thus could not synthesize odds ratios

### Odds ratios of DNA virus infection on ME/CFS

**Fig. 2:**
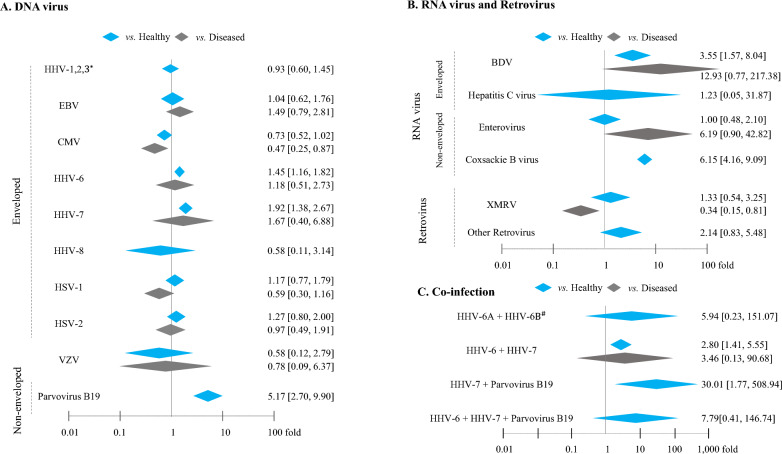
Meta-analysis for odds ratio of viral infections in ME/CFS. Ten DNA viruses (**A**), 5 RNA viruses(**B**) and 4 co-infections (**C**) were calculated by comparing between ME/CFS patients and healthy and/or diseased controls. *Meta-analysis was done together for three viruses; ^#^Original three studies observed HHV-6 infection by dividing into A and B subtype

Twelve species of DNA viruses were examined, and the odds ratios of viral infection in ME/CFS patients were investigated by comparison with healthy subjects and/or 4 different diseased subjects (Table 2). Meta-analyses showed that 2 viruses presented odds ratios greater than 2: 1.92 [95% CI 1.38–2.67] for HHV-7 and 5.50 [95% CI 2.70–9.90] for parvovirus B19 compared with only healthy subjects but not diseased subjects (Fig. 2A; Table 2).

### Odds ratios of RNA virus infections on ME/CFS

Four species of RNA viral infection in ME/CFS patients were compared with healthy subjects and/or 2 different diseased subjects. Both BDV and coxsackie B viruses presented odds ratios of 3.55 [95% CI 1.57–8.04] and 6.15 [95% CI 4.16–9.09], respectively, compared with healthy controls, while BDV (12.93 [95% CI 0.77–217.3]) and enterovirus (6.19 [95% CI 0.90–42.82] in random effect model) had odds ratios greater than 2 compared with subjects with disorders (such as neurological disease) (Fig. 2B; Table 2).

### Odds ratios of retrovirus infections on ME/CFS

Infection with retrovirus in ME/CFS patients showed an odds ratio of 2.14 [95% CI, 0.83–5.48] compared with healthy subjects. There were no significant odds ratios observed for XMRV patients when comparing them to either heathy or 7 different diseased subjects (Fig. 2B; Table 3). Only one study compared XMRV with patients with chronic fatigue, and the odds ratio was 2.35 [95% CI 0.12–45.32] (Table 3).

### Odds ratios of co-infections on ME/CFS

Four types of co-infections were compared between ME/CFS patients and healthy or chronic fatigue subjects. All types of co-infections showed a high odds ratio (at least 2.80 odds ratio) compared with any control type (Fig. 2C; Table 3). However, these data had an extremely wide confidence interval due to the limited number of studies.

## Discussion

There have been numerous attempts to determine the etiology of ME/CFS, and many hypotheses have been proposed. Given that the meaning of the name ME is related to viral infection and that PVFS is the official name of this disease in the ICD 10, viral infection has been continuously considered as a potential etiology of ME/CFS [25, 26]. Cluster outbreaks of ME/CFS in various regions and the potential connection to autoimmune reactions have led to suspicions of viral infections playing a role in the development of ME/CFS [27, 28]. While the discovery of the xenotropic murine leukemia virus (MLV)-related virus (XMRV) by Lombardi and colleagues in 2009 [29] brought disappointment, the suspicion of virus infection as an etiological factor has persisted. This is evident in the ongoing systematic review concerning HHV-6 in ME/CFS patients [30]. Furthermore, with the prevalence of substantial post-COVID patients suffering from unknown fatigue symptoms even after full recovery, this hypothesis of viral etiology is receiving renewed attention currently [31, 32].

To contribute to the investigation of the potential viral etiology, we analyzed previous research findings using a meta-analysis of odds ratios. A total of 64 studies investigated the infection rates of 18 species of viruses, including their co-infections, in ME/CFS patients and controls (Fig. 1; Table 1). Healthy subjects composed the control groups for all viruses and co-infections, while 11 species of viruses (including one co-infection) were examined in comparison with ME/CFS patients and subjects having one of following 10 diseases/disorders: multiple sclerosis, fibromyalgia, chronic fatigue, neurological disease, rheumatoid arthritis, transplants, HIV infection, neurological/muscular disease, prostate cancer, non-ME/CFS. The odds ratio is the most widely used parameter to quantify causal strength between two events in case control studies [33]; therefore, we examined the odds ratio to estimate the effect of viral infection on the risk of ME/CFS.

When we set an odds ratio ≥ 2.0 as the criterion for potentially risky virus infections, 2 DNA viruses (HHV-7 and parvovirus B19, including co-infection of HHV-6/7 plus parvovirus) and 3 RNA viruses (BDV, enterovirus and coxsackie B virus) showed an odds ratio greater than 2.0 against healthy and/or diseased subjects (Fig. 2A, B; Tables 2, 3). In fact, the difficulty of interpreting the OR has been a critical issue in many epidemiologic studies, and the reference point reflecting a “moderate association” odds ratio is 3.47 at a 1% disease prevalence rate (ME/CFS) in the nonexposed group [34]. Based on the well-known approximately 1% prevalence of ME/CFS in the general population, if we simultaneously apply a cutoff of ≥ 3.47 for the odds ratio for both healthy and diseased subjects, only BDV can meet this criterion, while the other viruses cannot meet this criterion due to the lack of data for diseased subjects (likely parvovirus B19, HHV-6/7 plus parvovirus and coxsackie B virus) or small odds ratio for healthy controls (enterovirus and HHV-6 plus HHV-7). However, BDV also has a limitation of substantial uncertainty, as shown by the wide range of the 95% confidence interval when comparing to diseased controls, which is primarily due to the use of only one dataset. In contrast, both parvovirus B19 and coxsackie B virus exhibited significantly elevated odds ratios with narrower confidence intervals, even though they were compared only to healthy controls (Fig. 2).

The viruses described above have been investigated to examine their contribution to human diseases without a clear etiology, such as ME/CFS. For example, parvovirus B19 can induce neurological manifestations, likely encephalitic symptoms [35, 36]. The moderate level of evidence was found regarding the association between BDV infection and ME/CFS among 169 Swedish CFS patients and 62 healthy controls [37]. More recently, one Chinese group suggested the involvement of BDV infection in the etiology of some neuropsychiatric disorders, including multiple sclerosis (25.0% prevalence) and CFS (12.7% prevalence) [38]. Some reports have revealed that multiple co-infections are correlated with the severity of signs and symptoms in ME/CFS patients [39, 40]. However, we cannot assume that multiple co-infections might potentially induce ME/CFS, as all co-infections were compared only with healthy control groups including subjects with chronic fatigue (Fig. 2C).

As we showed in our meta-analysis, however, any virus infection cannot satisfy the general conditions from the aspect of sensitivity and specificity. A group recently surveyed an infectious trigger and/or immune dysregulation by testing antibodies to 122 different pathogenic antigens and found no significant difference between 59 ME/CFS cases and 44 matched controls [41]. In our study, most of the diseased controls had multiple sclerosis (7 studies) and fibromyalgia (5 studies). ME/CFS, multiple sclerosis and fibromyalgia are likely female predominant diseases and share similar symptoms with ME/CFS, including a high fatigue prevalence of approximately 70 to 80% [42, 43]. It is worth noting that ME/CFS is a complex multisystem neurological disorder and is related to not only symptoms but also different pathogeneses and/or etiologies [44, 45]. Taken together, we could easily assume that any specific pathogenic infection will not be an etiology or single contributor to ME/CFS.

Along with continuous controversial evidence, certain viral infections are a contributor to pathogenesis or a trigger in the development of ME/CFS at least partially [46]. Viral infection-related immune dysregulations, such as immunosuppression or chronic inflammation due to immune complexes [47, 48], cytokine dysregulation [49], and autoimmunity [50], have been proposed in ME/CFS patients. Additionally, mitochondrial disruptions, characterized by altered adenosine triphosphate (ATP) levels and increased reactive oxygen species (ROS), have been observed in ME/CFS patients with chronic viral infections [51, 52]. Our present study has several limitations. These include the exclusion of non-English studies and data from two database resources, a lack of information regarding participant characteristics such as age and ethnicity, a relatively limited number of disease controls and recent data, and the absence of adjustment for potential confounders. The diverse methodologies and study populations have contributed to significant heterogeneity in the results. Furthermore, COVID-19 infection was not included in the current study due to insufficient time to determine its causal impact on ME/CFS. Well-designed large-scale studies will be essential to further investigate the potential role of viruses in the future.

## Conclusion

In conclusion, we have conducted a comprehensive assessment of the risk associated with viral infections in the etiology of ME/CFS. In contrast to previous studies, this research represents the first meta-analysis that systematically investigates many relevant viruses, and it has identified some potential viruses, including BDV, parvovirus B19, and coxsackie B virus. Despite certain limitations, our study provides valuable reference data for future research exploring virus-associated factors in ME/CFS.

### Supplementary Information

**Additional file 1: Figure S1. **Funnel plots.

**Additional file 2: Table S1**. Quality Assessment of 64 Studies using NOS.

**Additional file 3: Table S2**. Results of Egger’s test and assessment of heterogenicity.

## Data Availability

All data related to this study are available in the public domain.
