# Supplementary material for: Evaluation of viral infection as an etiology of ME/CFS: a systematic review and meta-analysis
Source: J Transl Med. 2023 Oct 28;21:763. doi: 10.1186/s12967-023-04635-0 (PMC10612276; doi:10.1186/s12967-023-04635-0)
Supplement: Supplementary file 1 — Additional file 1: Figure S1. Funnel plots. [file 12967_2023_4635_MOESM1_ESM.pptx]

## Slide 1
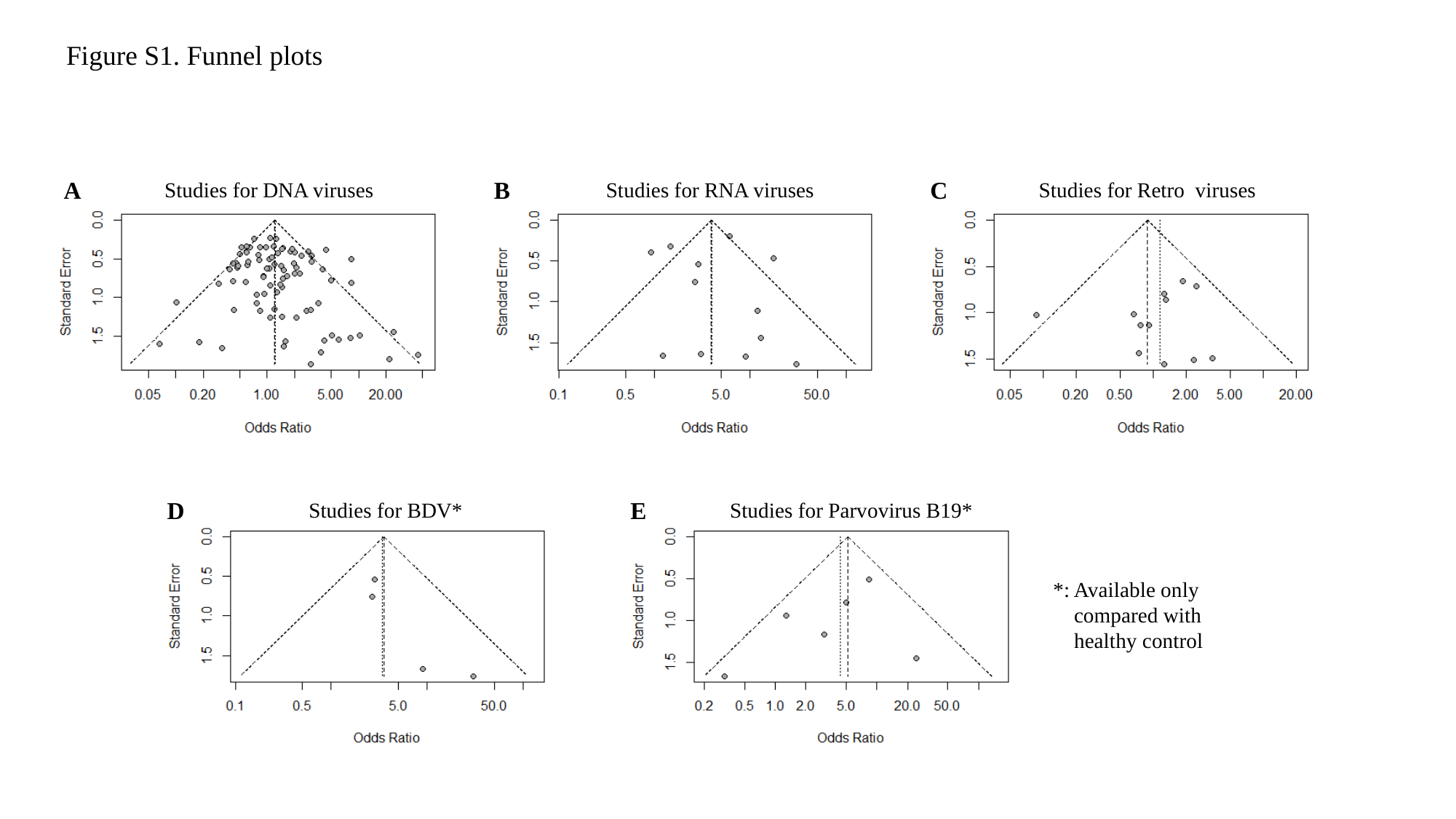

Figure S1. Funnel plots
B
Studies for RNA viruses
A
Studies for DNA viruses
C
Studies for Retro viruses
D
Studies for BDV*
E
Studies for Parvovirus B19*
*: Available only compared with healthy control
