# Supplementary material for: Evaluation of viral infection as an etiology of ME/CFS: a systematic review and meta-analysis
Source: J Transl Med. 2023 Oct 28;21:763. doi: 10.1186/s12967-023-04635-0 (PMC10612276; doi:10.1186/s12967-023-04635-0)
Supplement: Supplementary file 2 — Additional file 2: Table S1. Quality Assessment of 64 Studies using NOS. [file 12967_2023_4635_MOESM2_ESM.docx]

| **Table S1. Quality Assessment of 64 Studies using NOS** | | | |
| --- | --- | --- | --- |
| Publication year, *Country* /  First Author (Reference Number) | Studied Viruses  (N. of ME/CFS/Control subjects) | Total score (9 points)  (Selection/Comparability/Exposure) | |
| 2022, *Bulgaira* / Sabine Gravelsina [1] | HHV-6 (134/33 of Healthy control (HC)) | | **8** (4/1/3) |
| 2022, *United states* / Zheng Wang [2] | EBV, HHV-6 (166/83 of HC) | | **9** (4/2/3) |
| 2021, *United kingdom* / Ji-Sook Lee [3] | EBV, HHV-6,7, HSV-1 (30/76 of HC, 27 of Multiple sclerosis (MS)) | | **9** (4/2/3) |
| 2020, *Bulgaria* / Evelina Shikova [4] | EBV, CMV, HHV-6 (58/50 of HC) | | **8** (3/2/3) |
| 2020, *Germany* / Philipp Schreiner [5] | HHV-6 (25/10 of HC) | | **7** (3/1/3) |
| 2019, *United kingdom* / Jacqueline M Cliff [6] | EBV, CMV, HHV-6, HSV-1,2, VZV (250/107 of HC, 49 of MS) | | **9** (4/2/3) |
| 2019, *Sweden* / Jonas Blomberg [7] | HHV-1,2,3,6,7, EBV, CMV (65/76 of HC, 11 of Fibromyaligia (FM)) | | **8** (4/1/3) |
| 2017, *Germany* / Madlen Loebel [8] | EBV (50/50 of HC) | | **7** (3/1/3) |
| 2014, *Latvia* / Santa Rasa [9] | XMRV (150/30 of HC, 61 of Non-ME/CFS) | | **7** (3/1/3) |
| 2014, *United states* / Peter Gerondelis [10] | XMRV (72/37 of HC) | | **9** (4/2/3) |
| 2012, *United states* / Harvey J Alter [11] | XMRV (147/146 of HC) | | **9** (4/2/3) |
| 2012, *United states* / Peter D Burbelo [12] | HHV-6 (72/59 of HC) | | **7** (3/1/3) |
| 2012, *Italy* / F Maggi [13] | XMRV (65/25 of HC, 55 of FM, 25 of Rheumatoid arthritis (RA)) | | **8** (3/2/3) |
| 2012, *Latvia* / S. Chapenko [14] | HHV-6,7, Parvo B19 virus (108/90 of HC) | | **7** (3/1/3) |
| 2011, *Sweden* / Amal Elfaitouri [15] | XMRV (78/168 of HC) | | **5** (3/0/2) |
| 2011, *United states* / Brent C Satterfield [16] | XMRV (45/42 of HC) | | **7** (4/1/2) |
| 2011, *United states* / Clifford H Shin [17] | XMRV (100/200 of HC) | | **7** (4/0/3) |
| 2011, *Japan* / Furuta RA [18] | XMRV (100/500 of HC, 67 of Prostate cancer (PC)) | | **8** (3/2/3) |
| 2011, *Canada* / Imke Steffen [19] | XMRV (58/57 of HC) | | **8** (3/2/3) |
| 2011, *United kingdom* / Otto Erlwein [20] | XMRV (130/30 of HC) | | **6** (3/0/3) |
| 2011, *United states* / Brendan Oakes [21] | XMRV (112/36 of HC) | | **6** (3/0/3) |
| 2011, *United states* / Ali MA [22] | XMRV (61/9 of HC, 6 of Chronic fatigue (CF), 3 of Non-ME/CFS) | | **8** (3/2/3) |
| 2010, *United kingdom* / Lihan Zhang [23] | EBV, Enterovirus, Parvo B19 virus (117/29 of HC, 14 of Endogenous disease (ED)) | | **8** (3/2/3) |
| 2010, *United kingdom* / Jonathan R. Kerr [24] | Parvo B19 virus (200/200 of HC) | | **8** (3/2/3) |
| 2010, *United states* / Timothy J. Henrich [25] | XMRV (32/95 of HC, 97 of RA, 43 of HIV, 26 of Transplants) | | **7** (3/1/3) |
| 2010, *Germany* / Oliver Hohn [26] | XMRV (39/27 of HC, 112 of MS) | | **6** (3/1/2) |
| 2010, *United kingdom* / Harriet CT Groom [27] | XMRV (170/395 of HC) | | **6** (3/1/2) |
| 2010, *China* / Ping Hong [28] | XMRV (65/65 of HC, 20 of Non-ME/CFS) | | **8** (3/2/3) |
| 2010, *Netherland* / Frank J M van Kuppeveld [29] | XMRV (32/43 of HC) | | **9** (4/2/3) |
| 2010, *United states* / William M Switzer [30] | XMRV (51/56 of HC) | | **9** (4/2/3) |
| 2009, *Belgium* / MARC FRÉMONT [31] | EBV, HHV-6,7 Parvo B19 virus (48/35 of HC) | | **6** (3/0/3) |
| 2009, *Japan* / Yukiko Hakariya Kato [32] | Parvo B19 virus (58/49 of HC) | | **7** (3/1/3) |
| 2008*, United states* / J K S Chia [33] | CMV, Enterovirus (165/34 of Non-ME/CFS) | | **5** (3/0/2) |
| 2006, *Latvia* / S. Chapenko [34] | HHV-6,7 (17/20 of HC, 12 of CF) | | **7** (3/1/3) |
| 2003, *United states* / G. L. NICOLSON [35] | HHV-6 (200/100 of HC) | | **8** (3/2/3) |
| 2003, *Japan* / Kazufumi Ikuta [36] | EBV (44/38 of HC) | | **6** (3/0/3) |
| 2002, *United states* / David M Koelle [37] | EBV, CMV, HHV-6,7,8, HSV, Parvo B19 virus, VZV, JC virus, BK virus (22/22 of HC) | | **9** (4/2/3) |
| 2000, *United states* / D V Ablashi [38] | HHV-6,7,8 (35/28 of HC, 21 of MS, 20 of ND) | | **6** (3/0/3) |
| 2000, *United states* / W C Reeves [39] | HHV-6,7 (26/52 of HC) | | **9** (4/2/3) |
| 2000, *United states* / M Enbom [40] | HHV-6,8 (8/7 of Non-ME/CFS) | | **5** (2/0/3) |
| 2000, *United states* / I H Gelman [41] | Retrovirus (13/39 of Non-ME/CFS) | | **3** (3/0/0) |
| 1999, *United states* / H L Wallace 2^nd^ [42] | HHV-6,7 (76/73 of HC) | | **8** (3/2/3) |
| 1999, *Japan* / T Nakaya [43] | BDV (6/4 of HC) | | **6** (3/0/3) |
| 1999, *Sweden* / B Evengård [44] | BDV (169/30 of HC, 32 of Non-ME/CFS) | | **7** (3/1/3) |
| 1996*, Japan* / Teruo Kitani [45] | BDV (146/272 of HC) | | **7** (3/1/3) |
| 1996, *United kingdom* / A. McARDLE [46] | Enterovirus (54/10 of Non-ME/CFS) | | **5** (3/0/2) |
| 1995, *Japan* / T Sairenji [47] | EBV, HHV-6,7 (20/26 of HC) | | **6** (3/0/3) |
| 1995*, Italy* / D Di Luca [48] | HHV-6 (36), 7 (28/24 of HC) | | **9** (4/2/3) |
| 1994, *Netherland* / C M Swanink [49] | Enterovirus (76/76 of HC) | | **8** (3/2/3) |
| 1994, *United states* / W Heneine [50] | Retrovirus (21/21 of HC) | | **8** (3/2/3) |
| 1994, *United kingdom* / J W Gow [51] | Enterovirus (121/101 of Neurological/muscular disease (NMD)) | | **7** (2/2/3) |
| 1993, *Canada* / D Eymard [52] | EBV (17/7 of HC), CMV (14/7 of HC), HSV (9/4 of HC) | | **6** (2/1/3) |
| 1993, *Japan* / M Honda [53] | Retrovirus (30/30 of HC) | | **7** (3/1/3) |
| 1993, *United states* / Thomas M. Folks [54] | Retrovirus (26/28 of HC) | | **5** (1/1/3) |
| 1993, *United states* / A S Khan [55] | Retrovirus (21/42 of HC) | | **9** (4/2/3) |
| 1993, *United states* / WJ Gunn, PhD [56] | Retrovirus (68//68 of HC) | | **8** (3/2/3) |
| 1992, *Japan* / K Kawa [57] | EBV (10/28 of Non-ME/CFS) | | **8** (3/2/3) |
| 1992, *United states* / Paul H. Levine, MD [58] | HHV-6 (27/89 of HC), Retrovirus (31/105 of HC) | | **4** (1/0/3) |
| 1992, *United kingdom* / J W Gow [59] | Retrovirus (30/15 of HC, 15 of NMD) | | **7** (2/2/3) |
| 1992, *United kingdom* / C G Woodward [60] | EBV (101/94 of HC, 35 of CF) | | **8** (3/2/3) |
| 1991, *United states* / S. F. JOSEPHS [61] | HHV-6 (7/2 of HC) | | **6** (2/1/3) |
| 1991, *United states* / J K Dale [62] | Hepatitis C virus (36/14 of HC) | | **8** (3/2/3) |
| 1988, *United kingdom* / E J Bell [63] | Coxsackie B virus (290/500 of HC) | | **5** (1/1/3) |
| 1988, *United kingdom* / D Wakefield,A [64] | HHV-6 (59/15 of HC) | | **5** (1/1/3) |
| ^* :^ All studies are case-control study  N: Number, HC : Healthy control, MS : Multiple sclerosis, FM : Fibromyalgia, RA : Rheumatoid arthritis , CF : Chronic fatigue, ED : Endogenous depression, ND : Neurological disease, NMD : Neurological/muscular disease | | | |
|  | | | |

Suppl. References (64 studies included)

1. [Gravelsina S, Vilmane A, Svirskis S, Rasa-Dzelzkaleja S, Nora-Krukle Z, Vecvagare K, Krumina A, Leineman I, Shoenfeld Y, Murovska M: **Biomarkers in the diagnostic algorithm of myalgic encephalomyelitis/chronic fatigue syndrome**. *Frontiers in Immunology* 2022:5980.](https://pubmed.ncbi.nlm.nih.gov/36300129/)

2. [Wang Z, Waldman MF, Basavanhally TJ, Jacobs AR, Lopez G, Perichon RY, Ma JJ, Mackenzie EM, Healy JB, Wang Y: **Autoimmune gene expression profiling of fingerstick whole blood in Chronic Fatigue Syndrome**. *Journal of Translational Medicine* 2022, **20**(1):1-15.](https://pubmed.ncbi.nlm.nih.gov/36284352/)

3. [Lee J-S, Lacerda EM, Nacul L, Kingdon CC, Norris J, O'Boyle S, Palla L, Riley EM, Cliff JM: **Salivary DNA loads for human herpesviruses 6 and 7 are correlated with disease phenotype in myalgic Encephalomyelitis/Chronic fatigue syndrome**. *Frontiers in medicine* 2021:1129](https://pubmed.ncbi.nlm.nih.gov/34422848/).

4. [Shikova E, Reshkova V, Kumanova А, Raleva S, Alexandrova D, Capo N, Murovska M: **Cytomegalovirus, Epstein‐Barr virus, and human herpesvirus‐6 infections in patients with myalgic еncephalomyelitis/chronic fatigue syndrome**. *Journal of medical virology* 2020, **92**(12):3682-3688.](https://pubmed.ncbi.nlm.nih.gov/32129496/)

5. [Schreiner P, Harrer T, Scheibenbogen C, Lamer S, Schlosser A, Naviaux RK, Prusty BK: **Human herpesvirus-6 reactivation, mitochondrial fragmentation, and the coordination of antiviral and metabolic phenotypes in myalgic encephalomyelitis/chronic fatigue syndrome**. *Immunohorizons* 2020, **4**(4):201-215.](https://pubmed.ncbi.nlm.nih.gov/32327453/)

6. [Cliff JM, King EC, Lee J-S, Sepúlveda N, Wolf A-S, Kingdon C, Bowman E, Dockrell HM, Nacul L, Lacerda E: **Cellular immune function in myalgic encephalomyelitis/chronic fatigue syndrome (ME/CFS)**. *Frontiers in immunology* 2019:796.](https://pubmed.ncbi.nlm.nih.gov/31057538/)

7. [Blomberg J, Rizwan M, Böhlin-Wiener A, Elfaitouri A, Julin P, Zachrisson O, Rosén A, Gottfries C-G: **Antibodies to human herpesviruses in myalgic encephalomyelitis/chronic fatigue syndrome patients**. *Frontiers in Immunology* 2019, **10**:1946.](https://pubmed.ncbi.nlm.nih.gov/31475007/)

8. [Loebel M, Eckey M, Sotzny F, Hahn E, Bauer S, Grabowski P, Zerweck J, Holenya P, Hanitsch LG, Wittke K: **Serological profiling of the EBV immune response in Chronic Fatigue Syndrome using a peptide microarray**. *PloS one* 2017, **12**(6):e0179124.](https://pubmed.ncbi.nlm.nih.gov/28604802/)

9. [Rasa S, Nora-Krukle Z, Chapenko S, Krumina A, Roga S, Murovska M: **No evidence of XMRV provirus sequences in patients with myalgic encephalomyelitis/chronic fatigue syndrome and individuals with unspecified encephalopathy**. *The new microbiologica* 2014, **37**(1):17-24.](https://pubmed.ncbi.nlm.nih.gov/24531167/)

10. [Irlbeck DM, Vernon SD, McCleary KK, Bateman L, Klimas NG, Lapp CW, Peterson DL, Brown JR, Remlinger KS, Wilfret DA: **No association found between the detection of either xenotropic murine leukemia virus-related virus or polytropic murine leukemia virus and chronic fatigue syndrome in a blinded, multi-site, prospective study by the establishment and use of the SolveCFS BioBank**. *BMC Research Notes* 2014, **7**:1-10.](https://pubmed.ncbi.nlm.nih.gov/25092471/)

11. [Alter HJ, Mikovits JA, Switzer WM, Ruscetti FW, Lo S-C, Klimas N, Komaroff AL, Montoya JG, Bateman L, Levine S: **A multicenter blinded analysis indicates no association between chronic fatigue syndrome/myalgic encephalomyelitis and either xenotropic murine leukemia virus-related virus or polytropic murine leukemia virus**. *MBio* 2012, **3**(5):e00266-00212.](https://pubmed.ncbi.nlm.nih.gov/22991430/)

12. [Burbelo PD, Bayat A, Wagner J, Nutman TB, Baraniuk JN, Iadarola MJ: **No serological evidence for a role of HHV-6 infection in chronic fatigue syndrome**. *American journal of translational research* 2012, **4**(4):443.](https://pubmed.ncbi.nlm.nih.gov/23145212/)

13. [Maggi F, Bazzichi L, Sernissi F, Mazzetti P, Lanini L, Scarpellini P, Consensi A, Giacomelli C, Macera L, Vatteroni M: **Absence of xenotropic murine leukemia virus-related virus in Italian patients affected by chronic fatigue syndrome, fibromyalgia, or rheumatoid arthritis**. *International Journal of Immunopathology and Pharmacology* 2012, **25**(2):523-529.](https://pubmed.ncbi.nlm.nih.gov/22697086/)

14. [Chapenko S, Krumina A, Logina I, Rasa S, Chistjakovs M, Sultanova A, Viksna L, Murovska M: **Association of active human herpesvirus-6,-7 and parvovirus b19 infection with clinical outcomes in patients with myalgic encephalomyelitis/chronic fatigue syndrome**. *Advances in virology* 2012, **2012**.](https://pubmed.ncbi.nlm.nih.gov/22927850/)

15. [Elfaitouri A, Shao X, Mattsson Ulfstedt J, Muradrasoli S, Bölin Wiener A, Golbob S, Öhrmalm C, Matousek M, Zachrisson O, Gottfries C-G: **Murine gammaretrovirus group G3 was not found in Swedish patients with myalgic encephalomyelitis/chronic fatigue syndrome and fibromyalgia**. *PloS one* 2011, **6**(10):e24602.](https://pubmed.ncbi.nlm.nih.gov/22022360/)

16. [Satterfield BC, Garcia RA, Jia H, Tang S, Zheng H, Switzer WM: **Serologic and PCR testing of persons with chronic fatigue syndrome in the United States shows no association with xenotropic or polytropic murine leukemia virus-related viruses**. *Retrovirology* 2011, **8**:1-7.](https://pubmed.ncbi.nlm.nih.gov/21342521/)

17. [Shin CH, Bateman L, Schlaberg R, Bunker AM, Leonard CJ, Hughen RW, Light AR, Light KC, Singh IR: **Absence of XMRV retrovirus and other murine leukemia virus-related viruses in patients with chronic fatigue syndrome**. *Journal of virology* 2011, **85**(14):7195-720](https://pubmed.ncbi.nlm.nih.gov/21543496/)2.

18. [Furuta RA, Miyazawa T, Sugiyama T, Kuratsune H, Ikeda Y, Sato E, Misawa N, Nakatomi Y, Sakuma R, Yasui K: **No association of xenotropic murine leukemia virus-related virus with prostate cancer or chronic fatigue syndrome in Japan**. *Retrovirology* 2011, **8**(1):1-12.](https://pubmed.ncbi.nlm.nih.gov/21414229/)

19. [Steffen I, Tyrrell DL, Stein E, Montalvo L, Lee T-H, Zhou Y, Lu K, Switzer WM, Tang S, Jia H: **No evidence for XMRV nucleic acids, infectious virus or anti-XMRV antibodies in Canadian patients with chronic fatigue syndrome**. *PloS one* 2011, **6**(11):e27870.](https://pubmed.ncbi.nlm.nih.gov/22114717/)

20. [Erlwein O, Robinson MJ, Kaye S, Wills G, Izui S, Wessely S, Weber J, Cleare A, Collier D, McClure MO: **Investigation into the presence of and serological response to XMRV in CFS patients**. *PloS one* 2011, **6**(3):e17592.](https://pubmed.ncbi.nlm.nih.gov/21408077/)

21. [Oakes B, Qiu X, Levine S, Hackett J, Huber BT: **Failure to detect XMRV-specific antibodies in the plasma of CFS patients using highly sensitive chemiluminescence immunoassays**. *Advances in virology* 2011, **2011**.](https://pubmed.ncbi.nlm.nih.gov/22312356/)

22. [Ali MA, Dale JK, Kozak CA, Goldbach-Mansky R, Miller FW, Straus SE, Cohen JI: **Xenotropic murine leukemia virus-related virus is not associated with chronic fatigue syndrome in patients from different areas of the US in the 1990s**. *Virology Journal* 2011, **8**(1):1-8.](https://pubmed.ncbi.nlm.nih.gov/21943244/)

23. [Zhang L, Gough J, Christmas D, Mattey DL, Richards SC, Main J, Enlander D, Honeybourne D, Ayres JG, Nutt DJ: **Microbial infections in eight genomic subtypes of chronic fatigue syndrome/myalgic encephalomyelitis**. *Journal of clinical pathology* 2010, **63**(2):156-164.](https://pubmed.ncbi.nlm.nih.gov/19955554/)

24. [Kerr JR, Gough J, Richards SC, Main J, Enlander D, McCreary M, Komaroff AL, Chia JK: **Antibody to parvovirus B19 nonstructural protein is associated with chronic arthralgia in patients with chronic fatigue syndrome/myalgic encephalomyelitis**. *Journal of general virology* 2010, **91**(4):893-897.](https://pubmed.ncbi.nlm.nih.gov/20007355/)

25. [Henrich TJ, Li JZ, Felsenstein D, Kotton CN, Plenge R, Pereyra F, Marty FM, Lin NH, Grazioso P, Crochiere DM: **Xenotropic murine leukemia virus-related virus prevalence in patients with chronic fatigue syndrome or chronic immunomodulatory conditions**. *The Journal of infectious diseases* 2010, **202**(10):1478-1481.](https://pubmed.ncbi.nlm.nih.gov/20936980/)

26. [Hohn O, Strohschein K, Brandt AU, Seeher S, Klein S, Kurth R, Paul F, Meisel C, Scheibenbogen C, Bannert N: **No evidence for XMRV in German CFS and MS patients with fatigue despite the ability of the virus to infect human blood cells in vitro**. *PloS one* 2010, **5**(12):e15632.](https://pubmed.ncbi.nlm.nih.gov/21203514/)

27. [Groom HC, Boucherit VC, Makinson K, Randal E, Baptista S, Hagan S, Gow JW, Mattes FM, Breuer J, Kerr JR: **Absence of xenotropic murine leukaemia virus-related virus in UK patients with chronic fatigue syndrome**. *Retrovirology* 2010, **7**:1-10.](https://pubmed.ncbi.nlm.nih.gov/20156349/)

28. [Hong P, Li J, Li Y: **Failure to detect Xenotropic murine leukaemia virus-related virus in Chinese patients with chronic fatigue syndrome**. *Virology journal* 2010, **7**(1):1-9.](https://pubmed.ncbi.nlm.nih.gov/20836869/)

29. [van Kuppeveld FJ, de Jong AS, Lanke KH, Verhaegh GW, Melchers WJ, Swanink CM, Bleijenberg G, Netea MG, Galama JM, van der Meer JW: **Prevalence of xenotropic murine leukaemia virus-related virus in patients with chronic fatigue syndrome in the Netherlands: retrospective analysis of samples from an established cohort**. *Bmj* 2010, **340**.](https://pubmed.ncbi.nlm.nih.gov/20185493/)

30. [Switzer WM, Jia H, Hohn O, Zheng H, Tang S, Shankar A, Bannert N, Simmons G, Hendry RM, Falkenberg VR: **Absence of evidence of xenotropic murine leukemia virus-related virus infection in persons with chronic fatigue syndrome and healthy controls in the United States**. *Retrovirology* 2010, **7**:1-13.](https://pubmed.ncbi.nlm.nih.gov/20594299/)

31. [Fremont M, Metzger K, Rady H, Hulstaert J, De Meirleir K: **Detection of herpesviruses and parvovirus B19 in gastric and intestinal mucosa of chronic fatigue syndrome patients**. *In vivo* 2009, **23**(2):209-213.](https://pubmed.ncbi.nlm.nih.gov/19414405/)

32. [Kato YH, Yamate M, Tsujikawa M, Nishigaki H, Tanaka Y, Yunoki M, Kuratsune H, Watanabe Y, Ikuta K: **No apparent difference in the prevalence of parvovirus B19 infection between chronic fatigue syndrome patients and healthy controls in Japan**. *Journal of Clinical Virology* 2009, **44**(3):246-247.](https://pubmed.ncbi.nlm.nih.gov/19200778/)

33. [Chia JK, Chia AY: **Chronic fatigue syndrome is associated with chronic enterovirus infection of the stomach**. *Journal of clinical pathology* 2008, **61**(1):43-48.](https://pubmed.ncbi.nlm.nih.gov/17872383/)

34. [Chapenko S, Krumina A, Kozireva S, Nora Z, Sultanova A, Viksna L, Murovska M: **Activation of human herpesviruses 6 and 7 in patients with chronic fatigue syndrome**. *Journal of clinical virology* 2006, **37**:S47-S51.](https://pubmed.ncbi.nlm.nih.gov/17276369/)

35. [Nicolson G, Gan R, Haier J: **Multiple co‐infections (mycoplasma, chlamydia, human herpes virus‐6) in blood of chronic fatigue syndrome patients: association with signs and symptoms**. *Apmis* 2003, **111**(5):557-566.](https://pubmed.ncbi.nlm.nih.gov/12887507/)

36. [Ikuta K, Yamada T, Shimomura T, Kuratsune H, Kawahara R, Ikawa S, Ohnishi E, Sokawa Y, Fukushi H, Hirai K: **Diagnostic evaluation of 2′, 5′-oligoadenylate synthetase activities and antibodies against Epstein–Barr virus and Coxiella burnetii in patients with chronic fatigue syndrome in Japan**. *Microbes and infection* 2003, **5**(12):1096-1102.](https://pubmed.ncbi.nlm.nih.gov/14554250/)

37. [Koelle DM, Barcy S, Huang M-L, Ashley RL, Corey L, Zeh J, Ashton S, Buchwald D: **Markers of viral infection in monozygotic twins discordant for chronic fatigue syndrome**. *Clinical Infectious Diseases* 2002, **35**(5):518-525.](https://pubmed.ncbi.nlm.nih.gov/12173124/)

38. [Ablashi D, Eastman H, Owen C, Roman M, Friedman J, Zabriskie J, Peterson D, Pearson G, Whitman J: **Frequent HHV-6 reactivation in multiple sclerosis (MS) and chronic fatigue syndrome (CFS) patients**. *Journal of Clinical Virology* 2000, **16**(3):179-191.](https://pubmed.ncbi.nlm.nih.gov/10738137/)

39. [Reeves WC, Stamey FR, Black JB, Mawle AC, Stewart JA, Pellett PE: **Human herpesviruses 6 and 7 in chronic fatigue syndrome: a case-control study**. *Clinical infectious diseases* 2000, **31**(1):48-52.](https://pubmed.ncbi.nlm.nih.gov/10913395/)

40. [Enbom M, Linde A, Evengård B: **No evidence of active infection with human herpesvirus 6 (HHV-6) or HHV-8 in chronic fatigue syndrome**. *Journal of clinical microbiology* 2000, **38**(6):2457.](https://pubmed.ncbi.nlm.nih.gov/10917774/)

41. [Gelman IH, Unger ER, Mawle AC, Nisenbaum R, Reeves WC: **Chronic Fatigue Syndrome Is Not Associated With Expression of Endogenous Retrviral p15E**. *Molecular Diagnosis* 2000, **5**:155-156.](https://pubmed.ncbi.nlm.nih.gov/11066017/)

42. [Wallace HL, Natelson B, Gause W, Hay J: **Human herpesviruses in chronic fatigue syndrome**. *Clinical Diagnostic Laboratory Immunology* 1999, **6**(2):216-223.](https://pubmed.ncbi.nlm.nih.gov/10066657/)

43. [Nakaya T, Takahashi H, Nakamura Y, Kuratsune H, Kitani T, Machii T, Yamanishi K, Ikuta K: **Borna disease virus infection in two family clusters of patients with chronic fatigue syndrome**. *Microbiology and immunology* 1999, **43**(7):679-689.](https://pubmed.ncbi.nlm.nih.gov/10529109/)

44. [Evengård B, Briese T, Lindh G, Lee S, Lipkin WI: **Absence of evidence of Borna disease virus infection in Swedish patients with chronic fatigue syndrome**. *Journal of neurovirology* 1999, **5**(5):495-499.](https://pubmed.ncbi.nlm.nih.gov/10568886/)

45. [Kitani T, Kuratsune H, Fuke I, Nakamura Y, Nakaya T, Asahi S, Tobiume M, Yamaguti K, Machii T, Inagi R: **Possible correlation between Borna disease virus infection and Japanese patients with chronic fatigue syndrome**. *Microbiology and immunology* 1996, **40**(6):459-462.](https://pubmed.ncbi.nlm.nih.gov/8839433/)

46. [McArdle A, McArdle F, Jackson M, Page S, Fahal I, Edwards R: **Investigation by polymerase chain reaction of enteroviral infection in patients with chronic fatigue syndrome**. *Clinical Science* 1996, **90**(4):295-300.](https://pubmed.ncbi.nlm.nih.gov/8777836/)

47. [Sairenji T, Yamanishi K, Tachibana Y, Bertoni G, Kurata T: **Antibody responses to Epstein-Barr virus, human herpesvirus 6 and human herpesvirus 7 in patients with chronic fatigue syndrome**. *Intervirology* 1995, **38**(5):269-273.](https://pubmed.ncbi.nlm.nih.gov/8724857/)

48. [Di Luca D, Zorzenon M, Mirandola P, Colle R, Botta GA, Cassai E: **Human herpesvirus 6 and human herpesvirus 7 in chronic fatigue syndrome**. *Journal of clinical microbiology* 1995, **33**(6):1660-1661.](https://pubmed.ncbi.nlm.nih.gov/7650209/)

49. [Swanink CM, Melchers WJ, van der Meer JW, Vercoulen JH, Bleijenberg G, Fennis JF, Galama JM: **Enteroviruses and the chronic fatigue syndrome**. *Clinical infectious diseases* 1994, **19**(5):860-864.](https://pubmed.ncbi.nlm.nih.gov/7893870/)

50. [Heneine W, Woods TC, Sinha SD, Khan AS, Chapman LE, Schonberger LB, Folks TM: **Lack of evidence for infection with known human and animal retroviruses in patients with chronic fatigue syndrome**. *Clinical infectious diseases* 1994, **18**(Supplement_1):S121-S125.](https://pubmed.ncbi.nlm.nih.gov/8148438/)

51. [Gow J, Behan W, Simpson K, McGarry F, Keir S, Behan P: **Studies on enterovirus in patients with chronic fatigue syndrome**. *Clinical infectious diseases* 1994, **18**(Supplement_1):S126-S129.](https://pubmed.ncbi.nlm.nih.gov/8148439/)

52. [Eymard D, Lebel F, Miller M, Turgeon F: **Human herpesvirus 6 and chronic fatigue syndrome**. *Canadian Journal of Infectious Diseases and Medical Microbiology* 1993, **4**:199-202.](https://pubmed.ncbi.nlm.nih.gov/22346448/)

53. [Honda M, Kitamura K, Nakasone T, Fukushima Y, Matsuda S, Nishioka K, Matsuda J, Hashimoto N, Yamazaki S: **Japanese patients with chronic fatigue syndrome are negative for known retrovirus infections**. *Microbiology and immunology* 1993, **37**(10):779-784.](https://pubmed.ncbi.nlm.nih.gov/7507200/)

54. [Folks TM, Heneine W, Khan A, Woods T, And LC, Schonberger L: **Investigation of retroviral involvement in chronic fatigue syndrome**. In: *Ciba Foundation Symposium 173‐Chronic Fatigue Syndrome: Chronic Fatigue Syndrome: Ciba Foundation Symposium 173: 2007*: Wiley Online Library; 2007: 160-175.](https://pubmed.ncbi.nlm.nih.gov/8387909/)

55. [Khan AS, Heneine WM, Chapman LE, Gary HE, Woods TC, Folks TM, Schonberger LB: **Assessment of a retrovirus sequence and other possible risk factors for the chronic fatigue syndrome in adults**. *Annals of internal medicine* 1993, **118**(4):241-245.](https://pubmed.ncbi.nlm.nih.gov/8420441/)

56. [Control CfD, Prevention: **Inability of retroviral tests to identify persons with chronic fatigue syndrome, 1992**. *MMWR Morbidity and mortality weekly report* 1993, **42**(10):183-190.](https://pubmed.ncbi.nlm.nih.gov/8446093/)

57. [Kawai K, Kawai A: **Studies on the relationship between chronic fatigue syndrome and Epstein-Barr virus in Japan**. *Internal Medicine* 1992, **31**(3):313-318.](https://pubmed.ncbi.nlm.nih.gov/1319246/)

58. [Levine PH, Jacobson S, Pocinki AG, Cheney P, Peterson D, Connelly RR, Weil R, Robinson SM, Ablashi DV, Salahuddin SZ: **Clinical, epidemiologic, and virologic studies in four clusters of the chronic fatigue syndrome**. *Archives of internal medicine* 1992, **152**(8):1611-1616.](https://pubmed.ncbi.nlm.nih.gov/1323246/)

59. [Gow J, Simpson K, Schliephake A, Behan W, Morrison L, Cavanagh H, Rethwilm A, Behan P: **Search for retrovirus in the chronic fatigue syndrome**. *Journal of clinical pathology* 1992, **45**(12):1058-1061.](https://pubmed.ncbi.nlm.nih.gov/1479030/)

60. [Woodward C, Cox RA: **Epstein-Barr virus serology in the chronic fatigue syndrome**. *Journal of Infection* 1992, **24**(2):133-139.](https://pubmed.ncbi.nlm.nih.gov/1314860/)

61. [Josephs S, Henry B, Balachandran N, Strayer D, Peterson D, Komaroff A, Ablashi D: **HHV-6 reactivation in chronic fatigue syndrome**. *The Lancet* 1991, **337**(8753):1346-1347.](https://pubmed.ncbi.nlm.nih.gov/1674318/)

62. [Dale JK, Di Bisceglie AM, Hoofnagle J, Straus SE: **Chronic fatigue syndrome: lack of association with hepatitis C virus infection**. *Journal of medical virology* 1991, **34**(2):119-121.](https://pubmed.ncbi.nlm.nih.gov/1653818/)

63. [Bell E, McCartney R, Riding M: **Coxsackie B viruses and myalgic encephalomyelitis**. *Journal of the Royal Society of Medicine* 1988, **81**(6):329-331.](https://pubmed.ncbi.nlm.nih.gov/2841461/)

64. [Wakefield D, Lloyd A, Dwyer J, Salahuddin SZ, Ablashi D: **Human herpesvirus 6 and myalgic encephalomyelitis**. *The Lancet* 1988, **331**(8593):1059.](https://pubmed.ncbi.nlm.nih.gov/2896906/)
