# Supplementary material for: Evaluation of viral infection as an etiology of ME/CFS: a systematic review and meta-analysis
Source: J Transl Med. 2023 Oct 28;21:763. doi: 10.1186/s12967-023-04635-0 (PMC10612276; doi:10.1186/s12967-023-04635-0)
Supplement: Supplementary file 3 — Additional file 3: Table S2. Results of Egger’s test and assessment of heterogenicity. [file 12967_2023_4635_MOESM3_ESM.docx]

| **Table S2. Results of Egger’s test and assessment of heterogenicity** | | | | | |
| --- | --- | --- | --- | --- | --- |
| **Virus** | | **Control (N. for egger’s test/total data)** | | **p-value (Egger’s test)** | **Heterogenicity using I^2^** |
| DNA virus | HHV-1,2,3 | | Healthy (1/1) | 0.3853 | 0% |
|  | EBV (HHV-4) | | Healthy (13/14) | 0.2160 | 52% |
|  |  |  | Diseased (3/4) | 0.5896 | 0% |
|  | CMV (HHV-5) | | Healthy (6/6) | 0.1818 | 0% |
|  |  |  | Diseased (2/3) | NA | 0% |
|  | HHV-6 | | Healthy (26/26) | 0.8523 | 42% |
|  |  |  | Diseased (4/4) | 0.4448 | 0% |
|  | HHV-7 | | Healthy (11/11) | 0.6949 | 0% |
|  |  |  | Diseased (1/1) | NA | NA |
|  | HHV-8 | | Healthy (2/3) | NA | 7% |
|  | HSV-1 | | Healthy (3/3) | 0.6405 | 0% |
|  |  |  | Diseased (1/1) | NA | NA |
|  | HSV-2 | | Healthy (2/2) | NA | 0% |
|  |  |  | Diseased (1/1) | NA | NA |
|  | VZV | | Healthy (1/2) | NA | NA |
|  |  |  | Diseased (1/1) | NA | NA |
|  | Parvovirus B19 | | Healthy (6/7) | 0.0201 | 32% |
| Total DNA viruses | | | (85/92) | **0.1926** | **41%** |
| RNA virus | BDV | | Healthy (4/4) | 0.0784 | 0% |
|  |  |  | Diseased (1/1) | NA | NA |
|  | Hepatitis C virus | | Healthy (1/1) | NA | NA |
|  | Enterovirus | | Healthy (2/2) | NA | 0% |
|  |  |  | Diseased (3/4) | 0.6726 | 90% |
|  | Coxsackie B virus | | Healthy (1/1) | NA | NA |
| Total RNA viruses | | | (12/13) | **0.9891** | **74%** |
| Retrovirus | XMRV | | Healthy (5/24) | 0.7493 | 0% |
|  |  |  | Diseased (5/13) | 0.0161 | 21% |
|  | Other Retrovirus | | Healthy (2/8) | NA | 0% |
| Total Retroviruses | | | (12/46) | **0.0682** | **0%** |
| Co-infection | HHV-6A + HHV-6B | | Healthy (1/1) | NA | NA |
|  | HHV-6 + HHV-7 | | Healthy (3/3) | 0.4827 | 32% |
|  |  |  | Diseased (1/1) | NA | NA |
|  | HHV-7 + Parvovirus B19 | | Healthy (1/1) | NA | NA |
|  | HHV-6 + HHV-7 Parvovirus B19 | | Healthy (1/1) | NA | NA |
| NA: It is not applicable for Egger’s test due to less than three available data or for heterogeneity using I^2^. | | | | |  |
